# Hourly Seamless Surface O_3_ Estimates by Integrating the Chemical Transport and Machine Learning Models in the Beijing-Tianjin-Hebei Region

**DOI:** 10.3390/ijerph19148511

**Published:** 2022-07-12

**Authors:** Wenhao Xue, Jing Zhang, Xiaomin Hu, Zhe Yang, Jing Wei

**Affiliations:** 1 School of Economics, Qingdao University, Qingdao 266071, China; xuewh@mail.bnu.edu.cn (W.X.); yz69env@163.com (Z.Y.); 2 College of Global Change and Earth System Science, Beijing Normal University, Beijing 100875, China; 201821490005@mail.bnu.edu.cn; 3 Department of Atmospheric and Oceanic Science, Earth System Science Interdisciplinary Center, University of Maryland, College Park, MD 20742, USA

**Keywords:** hourly ozone, Beijing-Tianjin-Hebei, random forest, WRF-Chem, air pollution

## Abstract

Surface ozone (O_3_) is an important atmospheric trace gas, posing an enormous threat to ecological security and human health. Currently, the core objective of air pollution control in China is to realize the joint treatment of fine particulate matter (PM_2.5_) and O_3_. However, high-accuracy near-surface O_3_ maps remain lacking. Therefore, we established a new model to determine the full-coverage hourly O_3_ concentration with the WRF-Chem and random forest (RF) models combined with anthropogenic emission data and meteorological datasets. Based on this method, choosing the Beijing-Tianjin-Hebei (BTH) region in 2018 as an example, full-coverage hourly O_3_ maps were generated at a horizontal resolution of 9 km. The performance evaluation results indicated that the new model is reliable with a sample (station)-based 10-fold cross-validation (10-CV) R^2^ value of 0.94 (0.90) and root mean square error (RMSE) of 14.58 (19.18) µg m^−3^. In addition, the estimated O_3_ concentration is accurately determined at varying temporal scales with sample-based 10-CV R^2^ values of 0.96, 0.98 and 0.98 at the daily, monthly, and seasonal scales, respectively, which is highly superior to traditional derivation algorithms and other techniques in previous studies. An initial increase and subsequent decrease, which constitute the diurnal variation in the O_3_ concentration associated with temperature and solar radiation variations, were captured. The highest concentration reached approximately 112.73 ± 9.65 μg m^−3^ at 15:00 local time (1500 LT) in the BTH region. Summertime O_3_ posed a high pollution risk across the whole BTH region, especially in southern cities, and the pollution duration accounted for more than 50% of the summer season. Additionally, 43 and two days exhibited light and moderate O_3_ pollution, respectively, across the BTH region in 2018. Overall, the new method can be beneficial for near-surface O_3_ estimation with a high spatiotemporal resolution, which can be valuable for research in related fields.

## 1. Introduction

Since the 21st century, China has experienced a period of rapid economic growth, urbanization, and industrialization. By 2020, the annual gross domestic product (GDP) reached approximately 101,598.62 billion yuan, ranking second globally (China Statistical Yearbook). However, the industrial structure in the early 21st century was extensive and mainly depended on energy, raw materials, and labor, resulting in the large release of air pollutants into the atmosphere [1,2,3]. These air pollutants exert a notable impact on human health, vegetation growth, and environmental change [4,5,6]. It was estimated that the number of fine particulate matter (PM_2.5_)-related deaths in China increased by approximately 390,000 from 2002 to 2017 [2]. Therefore, air pollution control has become one of the primary tasks of government environmental management at local and national scales. Fortunately, since 2012, China has conducted extensive real-time monitoring of six conventional pollutants [7], including PM_2.5_, coarse particulate matter (PM_10_), nitrogen dioxide (NO_2_), sulfur dioxide (SO_2_), carbon monoxide (CO), and ozone (O_3_). Then, with the proposal of an action plan for air pollution prevention and control by the Chinese government on September 10, 2013, the air quality has generally improved, especially in terms of the PM_10_ and PM_2.5_ concentrations, and notable decreasing trends have been captured throughout mainland China [8,9,10]. Despite all these achievements, the near-surface O_3_ concentration has exhibited the opposite trend to that of the particulate matter concentration [11,12].

O_3_ is one of the main secondary air pollutants in China and is formed by volatile organic compounds (VOCs) and nitrogen oxides (NO_x_) under solar radiation-driven reaction conditions [13]. According to epidemiological studies, the morbidity and mortality of respiratory diseases, heart diseases and even cancer are closely related to O_3_ loading levels [14,15,16]. In addition, high O_3_ loadings can destroy the vegetation physiological structure and growth environment, resulting in crop reduction and ultimately affecting food prices. Recently, several O_3_ pollution episodes have occurred in China, especially in urban agglomeration areas, and the annual mean daily maximum 8-h (MDA8) O_3_ concentration reached approximately 193, 170 and 165 μg m^−3^ across the Beijing-Tianjin-Hebei (BTH), Yangtze River Delta (YRD) and Pearl River Delta (PRD) regions, respectively, in 2017 [17]. Therefore, many studies have been carried out on ozone pollution at local and national scales, including field measurements of the ozone concentration [18,19], determination of the relationship among ozone precursors [20,21], analysis of the influences of meteorology on ozone pollution episodes [22,23], and attribution of emission sources [24]. However, previous studies have mostly considered surface observation records, which exhibit a discontinuous spatial distribution. In addition, due to the extremely high cost of manpower and material resources associated with ground-based monitoring, ozone concentration records are often discontinuous over time. Therefore, full-coverage high-quality near-surface O_3_ datasets are urgently needed for future studies on environmental economics, epidemiology, and climate change.

Two methods are generally employed to estimate the O_3_ concentration: model simulation and algorithm inversion. In regard to model simulation, regional air quality prediction models have been extensively adopted, e.g., the Community Multiscale Air Quality Modeling System (CMAQ), global chemical transport models (GEOS-Chem), Nested Air Quality Prediction Modeling System (NAQPMS) and Weather Research and Forecasting-Chemistry (WRF-Chem) model. Lu et al. (2019) applied the GEOS-Chem model to map the spatial distribution of the MDA8 O_3_ concentration from May to August from 2016–2017 across China, and the spatial correlation coefficient (R^2^) value reached approximately 0.67 [25]. Relying on the WRF-Chem model, Li et al. (2020) estimated the hourly O_3_ concentration in summer across the Lanzhou region [26]. Compared to surface measurements, the R^2^ values at each station were all consistently less than 0.4. Then, based on the above models, the formation, transport, and dissipation of O_3_ could be explained in detail via mechanism analysis [27,28,29]. However, deviations occurred from emission inventories, resulting in many uncertainties in O_3_ estimation. With the improvement of traditional statistics and the development of mathematical algorithms, the aforementioned problem has been increasingly resolved. Among the various techniques, traditional statistical models, e.g., multiple linear regression (MLR), linear mixed effect model (LME), geographically weighted regression (GWR), land use regression (LUR) and generalized additive model (GAM), have been widely implemented to estimate the concentration of air pollutants [30,31,32]. Zhang et al. (2020) adopted a GWR model to estimate the monthly O_3_ concentration in eastern China, and the validation based R^2^ value reached approximately 0.77 [33]. Thereafter, due to their strong data mining and information capture abilities, machine/deep learning-based methods have increasingly replaced traditional statistical methods. Zhan et al. (2018) selected the random forest (RF) model to estimate the MDA8 O_3_ concentration in 2015 across China with a cross-validation R^2^ value of 0.69 [34]. Then, based on the extreme gradient boosting (XGBoost) algorithm, the daily O_3_ concentration was simulated at the national scale, and the cross-validation R^2^ value reached 0.78 [22]. In addition, other machine learning methods have been widely applied to derive O_3_ and other air pollutant concentrations at national and local scales [35,36]. Despite all these developments, the estimation accuracy remains low, and high uncertainties still persist in previous algorithms. Furthermore, simulation of the O_3_ concentration in most previous studies occurred at a coarse temporal resolution, i.e., monthly, and daily resolutions, which cannot meet the requirements of meticulous research on short-term ozone pollution episodes. Therefore, accurate inversion of the hourly O_3_ concentration is urgently needed for environmental governance and policy implementation purposes.

Here, our objective is to establish an advanced approach to determine the full-coverage hourly accurate near-surface ozone concentration. Throughout all of mainland China, as one of the leading political, economic, and cultural centers, the BTH region is typically exposed to the highest O_3_ pollution burden. Therefore, this region was selected as an example in this study. For this purpose, the WRF-Chem model was combined with the RF method, meteorological factors, and other ancillary datasets to simulate the hourly O_3_ concentration across the whole BTH region from 1 January 2018, 00:00 local time (0000 LT) to 31 December 2018, 23:00 local time (2300 LT) at a horizontal resolution of 9 km × 9 km. In addition, we compared our algorithm to other similar algorithms and studies. Based on this approach, an hourly ozone map was established covering the BTH region, and we further performed a comprehensive investigation of the spatial distribution of the O_3_ concentration and ozone pollution level in the BTH region.

## 2. Materials and Methods

### 2.1. Study Area

In this study, the hourly O_3_ concentration in the BTH region is estimated. This region is located in northern China at latitudes and longitudes ranging from 36.0° N–42.6° N and 113.5° E–119.8° E, respectively. This region covers an area of approximately 218,000 km^2^ and includes Beijing (BJ) and Tianjin (TJ) and 11 cities in Hebei Province (Baoding (BD), Cangzhou (CZ), Chengde (CD), Handan (HD), Hengshui (HS), Langfang (LF), Qinhuangdao (QHD), Shijiazhuang (SJZ), Tangshan (TS), Xingtai (XT) and Zhangjiakou (ZJK)). In addition, this area hosts more than 8% of the population of China. As one of the large urban agglomerations in China, regional industrialization, urbanization, and motorization are closely related to changes in the atmospheric environment, thus forming a symbiotic situation among the emissions of coal fires, motor vehicles, and industrial exhaust. Especially regarding the emissions of O_3_ precursors, i.e., NO_x_ and VOCs, a notable increasing trend has been captured in recent years, resulting in heavy ozone pollution in this region [16]. High ozone loading also seriously affects the health of residents. When the MDA8 O_3_ concentration met the Chinese Ambient Air Quality Standards (CAAQS) Grade II standard, an increasing of 10 μg m^−3^ O_3_ concentration could lead to about a 0.31% increase in daily emergency room visits in Beijing [37]. In addition, a nonlinear association was exited between ozone and ischemic stroke, and younger adults are more susceptible to extremely high ozone levels than the elderly population in Beijing [38].

### 2.2. Datasets

#### 2.2.1. Measured Near-Surface Ozone

Hourly near-surface O_3_ records were collected from 87 state-managed environmental real-time monitoring stations across the BTH region. Figure S1 shows the spatial distribution of the O_3_ monitoring stations. In general, all cities included more than three sites. Beijing and Tianjin contained the most stations, with 12 and 20 monitoring stations, respectively. In this study, observation records from 0000 LT (GMT+8) on 1 January 2018, to 2300 LT on 31 December 2018, were collected as training samples and validation datasets. Moreover, to prevent systematic errors caused by the monitoring processes, observation records exceeding three times the standard deviation were eliminated. In addition, to avoid instrument failure, any values remaining constant for three consecutive hours were removed [39]. In regard to the state-managed environmental real-time monitoring stations, the O_3_ concentration was measured via the ultraviolet spectrophotometry method. However, the air quality monitoring protocol was amended on 1 September 2018 [40]. Therefore, we transformed the observed concentrations before this date via multiplication with a fixed coefficient, which was approximately 0.92 [41]. An uneven spatial distribution of the measurement stations occurs in the BTH region, resulting in multiple stations existing in the same grid. Therefore, we calculated the average concentration if one grid contained multiple records. Eventually, 289,553 effective hourly O_3_ records were collected for modeling.

#### 2.2.2. WRF-Chem-Simulated Ozone

In our two-stage model, the WRF-Chem model version 3.9.1 (WRF-Chem 3.9.1) was applied to simulate the O_3_ concentration with temporal and spatial resolutions of 1 h and 9 km, respectively, at the first stage [42]. In regard to the WRF-Chem model, meteorological and emission data are the essential driving factors of the initial field and boundary conditions. Meteorological data were collected from the National Centers for Environmental Prediction (NCEP) Final Operational Global Analysis dataset with temporal and spatial resolutions of 6 h and 1° × 1°, respectively. In terms of the adopted emission datasets, anthropogenic and biogenic emissions inventory data were obtained from the China Multiresolution Emission Inventory (MEIC) and Model of Emissions of Gases and Aerosols from Nature (MEGAN) at horizontal and temporal resolutions of 0.25° × 0.25° and 1 month, respectively [43]. To ensure temporal consistency, all emission driving datasets were interpolated to the hourly scale.

#### 2.2.3. Meteorological Factors

Ozone formation is greatly limited by meteorological conditions [44]. Based on this consideration, eight meteorological factors, i.e., the 2-m temperature (TEM, unit: K), 10-m wind speed (WS, unit: m s^−1^) and wind direction (WD, unit: degree), solar radiation (RAD, unit: W m^−2^), boundary layer height (BLH, unit: m), surface pressure (SP, unit: kPa), relative humidity (RH, unit: %) and total evaporation (EVA, unit: mm) were selected to reflect the generation, transport and dissipation processes of O_3_. However, most currently available meteorological datasets do not reach the hourly temporal resolution. Fortunately, fifth-generation European Centre for Medium-Range Weather Forecasts (ECMWF) reanalysis products (ERA5) have been released since 2018 (www.ecmwf.int), and the temporal resolutions have been increased to hourly intervals. Moreover, we interpolated the spatial resolutions of the above meteorological factors from 0.25° × 0.25° to 9 km with the bilinear interpolation method to ensure data consistency.

#### 2.2.4. Other Ancillary Data

Vegetation can release biogenic volatile organic compounds (BVOCs), which are also an important precursor of ozone generation [45]. Here, we collected annual cover vegetation data (CVL, the sum of low and high cover vegetation levels) sourced from the ERA5 land version dataset on a single level at a horizontal resolution of 0.25° × 0.25°. In addition, the hourly vertical integral of the divergence in the ozone flux (VIDO) was retrieved from the ERA5 product to reflect the ozone loading. Similar to meteorological factors, the above two parameters are both resampled to the same spatial resolution as that of the WRF-Chem-simulated O_3_ concentration.

### 2.3. Methodology

#### 2.3.1. Two-Stage Model

**Figure 1:**
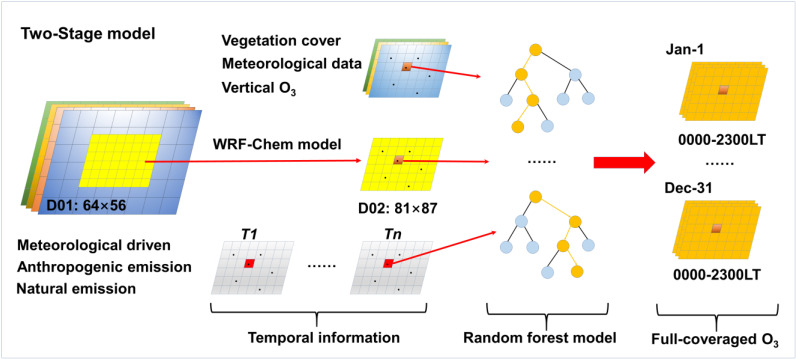
Flowchart of the two-stage model.

To estimate the full-coverage near-surface O_3_ concentration in the BTH region, a two-stage model was established in this study. Figure 1 shows a flowchart of our two-stage model. At the first stage, the WRF-Chem model was employed to explain the generation processes of O_3_. In addition, the impacts of anthropogenic and natural emissions on O_3_ concentrations were revealed. The WRF model is a mesoscale numerical simulation and data assimilation system that can simulate physical processes at cloud and weather scales [46]. Moreover, to improve the simulation accuracy, three- and four-dimensional variational assimilation algorithms and multilayer nesting grids were adopted in the latest version of the WRF model. To reveal the chemical processes of ozone, we adopted the online coupled chemical transport module of the WRF model, i.e., the WRF-Chem model [42]. For this model, the meteorological conditions and chemical composition can be synchronously and completely simulated, which exhibit the same time step, simulation areas, spatial resolution, and vertical coordinates, in addition to realizing two-way feedback simulation of atmospheric and chemical substances in real-time. In addition, this model can be employed for the simulation of the emission and transportation of atmospheric chemical components, and the interaction between gaseous pollutants (O_3_ and NO_2_) and particulate matter (PM_2.5_ and PM_10_) can be exactly captured. In this study, a double nesting grid was built with the Lambert projection at the first stage of our 2-stage model. In regard to the first-level domain, a grid with a size of 64 × 56 was established at a horizontal resolution of 27 km × 27 km, which could cover most of North China. Then, to further improve the simulation accuracy and resolution, the second domain (D02) was established based on the results of the first domain at a horizontal resolution of 9 km × 9 km (size: 81 × 87), which could cover the whole BTH region. In addition, for the purpose of physical mechanism unification, both grids were established under the same configurations as the boundary layer scheme of Yonsei University [47], Noah land surface scheme [48], Grell three-dimensional cumulus parameterization scheme [49] and Morrison double-moment microphysics scheme. Moreover, the radiation transport scheme was unified between the two domains, and the Goddard [50] and rapid radiative transfer models [51] were selected for the shortwave and longwave radiation schemes, respectively, of the WRF-Chem model. In addition, the chemical mechanism was consistent between the two domains, which entailed the Carbon-Bond Mechanism version Z [52]. In terms of the simulation of the near-surface O_3_ concentration, due to the inherent limitations of the WRF-Chem model, a monthly simulation cycle was set. Before simulation in each month, 48-h spin-up processes were first conducted for model preheating. Through the WRF-Chem model, the hourly ozone concentration was preliminarily simulated from 1 January 2018 to 31 December 2018, across the BTH region.

However, because notable spatiotemporal heterogeneities exist in O_3_ concentration data, deviations are still found when only employing the WRF-Chem model in the first stage. Therefore, we selected the RF model [53] to further determine the relationship between O_3_ and various independent variables at the second stage to improve the O_3_ inversion accuracy with Equation (1).

(1) $$O_{3\_}Pre_{i,j,h}=f_{\mathrm{RF}}\;\left[\mathrm{WRF}O_{3_{i,j,h}},\;{\mathrm{TEMI}}_{i,j,h},\;{\mathrm{TEM}}_{i,j,h},\;{\mathrm{RAD}}_{i,j,h},\ldots ,\;{\mathrm{RH}}_{i,j,h},{\mathrm{CVL}}_{i,j,h}\right]$$

where $O_{3\_}Pre_{i,j,h}$ denotes the simulated near-surface O_3_ concentration in grid *i* on day *j* at hour *h*, $\mathrm{WRF}O_{3_{i,j,h}}$ denotes the WRF-Chem simulated near-surface O_3_ concentration (WRFO_3_) in grid *i* on day *j* at hour *h* and ${\mathrm{TEMI}}_{i,j,h}$ denotes the temporal information in grid *i* on day *j* at hour *h*. In this study, a temporal weighted matrix was established according to the method described by Xue et al., which includes the day of the year (DOY), time distance of one day to spring, summer, autumn and winter, and local time (LT) [54]. In addition to temporal information, VIDO, CVL and meteorological parameters, including TEM, RAD, WS, WD, BLH, SP, EVA, and RH, in grid *i* on day *j* at hour *h* were selected as explanatory variables for model construction. To ensure that all input factors could attain statistical significance and avoid multicollinearity, correlation analysis and collinearity diagnosis methods were adopted here. Table S1 lists the correlation coefficient (R) and variance inflation factor (VIF) between the surface measured O_3_ concentration and all independent variables used for modeling in 2018 across the BTH region. Overall, WRFO_3_, TEM, RAD, WD, BLH and CVL imposed significant positive effects on O_3_ (*p* < 0.01). Among these variables, except for WRFO_3_, the highest R value of 0.66 was attained by TEM. The O_3_ concentration exhibited a significant negative response to TEMI, WS, SP, EVA, RH and VIDO, with R ranging from −0.08 to −0.56 (*p* < 0.01). Moreover, according to the threshold proposed by Ziegel et al., if the VIF value is higher than 10, significant collinearity exists among the variables [55]. In our model, the VIF values of all variables were lower than 4 (ranging from 1.04 to 3.91), which suggests that no multicollinearity occurred in the input data of the RF model. Thus, significant interrelation and no multicollinearity indicated that all independent variables selected in this paper could be considered in O_3_ concentration estimation in 2018 across the BTH region.

Before hourly O_3_ simulation, we also evaluated the contribution of all independent variables. Table 1 also lists the feature importance (FI) of all input datasets of our two-stage model. The total FI value is 100%, which reflects the contributions of each independent variable to model training, and a higher FI value indicates a higher contribution of the RF model to O_3_ estimation. The highest contribution was yielded by WRFO_3,_ with an FI value of 59.2%. And the second major contributing factor was TEM, with an FI value of 14.1%. Generally, a high temperature facilitates the volatilization of VOCs, and heavy O_3_ pollution episodes usually occur at high temperatures [56]. In addition, high temperatures can affect atmospheric turbulence and accelerate photochemical reactions [57]. Figure S1 also shows the mean temperature in the BTH region. In general, the mean temperature reaches approximately 283.75 K, and a high temperature was captured in the southern BTH region. Another vital reaction condition is radiation, which accounts for ~6.6% of the estimated hourly O_3_ concentration. Radiation is a necessary condition for photochemical reactions, which could limit the release of biological VOCs and the photodissociation reaction rate, resulting in O_3_ loading changes. The next important contributing factor was RH (FI value: ~5.4), which can affect radiation transfer, reduce the air temperature, and accelerate O_3_ dissipation. Moreover, the total contribution of the considered temporal information and other meteorological factors reached approximately 14.7%, and these factors could describe the temporal variation, generation, transport, and dissipation processes of O_3_ across the BTH region.

In addition, many traditional models used for O_3_ concentration estimation were selected for training based on the same input datasets as those employed for our two-stage model for comparison purposes, including MLR, LME, GWR, GAM and traditional two-stage models.

#### 2.3.2. Evaluation Approach

Two tenfold cross-validation (10-CV) approaches, i.e., sample- and station-based 10-CV approaches, were selected to evaluate the simulated results of our two-stage model. In regard to the sample-based 10-CV approach, the total samples (the explained variable is the measured O_3_, the explanatory variables include WRF-Chem-simulated O_3_, meteorological factors and other ancillary data, and each explanatory variable record corresponds to 12 explanatory variables) were randomly divided into ten groups according to the data samples. Among all partitions, nine partitions were selected as training samples for modeling with the two-stage model, while the remaining samples were adopted as the testing dataset. The above process was repeated ten times to ensure that all samples were applied for one time testing and nine times modeling. In the station-based 10-CV approach, the total samples were divided into ten subsets according to the O_3_ monitoring stations. Similarly, all samples were considered nine times for training and once for testing. Moreover, various statistical indexes, including regression equation parameters (slope and intercept), goodness of fit (R^2^), root mean square error (RMSE) and mean absolute error (MAE), were employed to evaluate the consistency between the simulated and observed O_3_ concentrations.

## 3. Results

### 3.1. Overall Accuracy Evaluation

**Figure 2:**
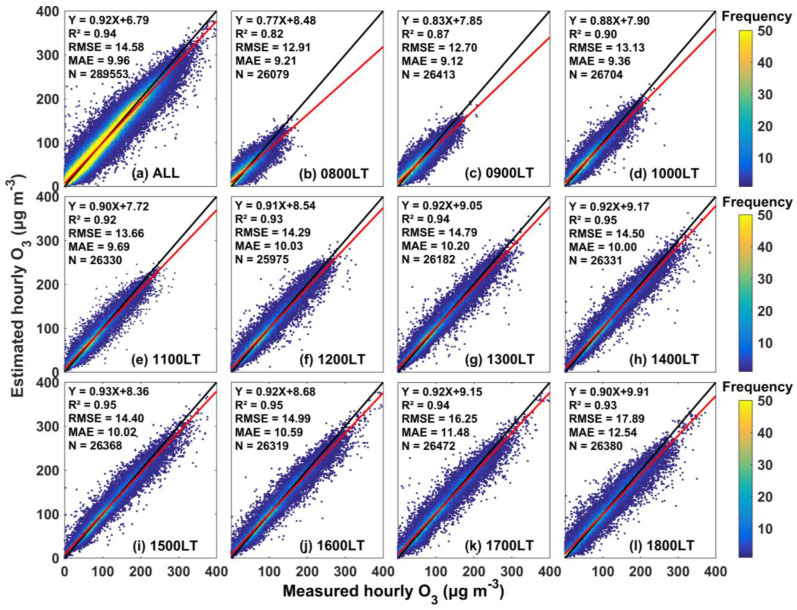
Density scatter plots of the sample-based 10-CV results from 08:00 local time (0800 LT) to 18:00 local time (1800 LT) across the BTH region in 2018: (**a**) all hourly records from 0800 to 1800 LT; and (**b**–**l**) sample-based 10-CV values for each hour from 0800 to 1800 LT. The black lines denote 1:1 lines and the red lines denote linear regression fitting lines.

Generally, the two-stage model achieved a strong data-mining ability. Figure 2 shows the sample-based 10-CV results of our model in terms of O_3_ estimation on an hourly basis across the BTH region. Because ozone formation occurs under solar radiation, we only selected the results from 08:00 local time (0800 LT) to 1800 LT for illustration. Overall, our model could establish relationships between the hourly measured O_3_ concentration and independent variables with overall coefficient values of R^2^, RMSE and MAE of 0.94, 14.58 μg m^−3^ and 9.96 μg m^−3^, respectively. In addition, our model greatly avoided overfitting, and the slope of the best-fit linear regression lines reached 0.92. In addition, the evaluation indexes at each hour were calculated. The two-stage model was highly accurate in hourly O_3_ concentration simulation, with high sample-based 10-CV R^2^ values and linear regression slopes ranging from 0.82 to 0.95 and 0.77 to 0.93, respectively. In addition, the uncertainties in the two-stage model were low with a linear regression intercept, RMSE and MAE ranging from 7.72–9.91 μg m^−3^, 12.70–17.89 μg m^−3^ and 9.12–12.54 μg m^−3^, respectively. However, the reaction conditions of O_3_, such as radiation and temperature, are different throughout the day, while the human activity and precursor concentration levels also vary, resulting in the evaluation indexes exhibiting slight differences throughout the day. Better estimation performances were captured from 1200 to 1800 LT. The R^2^ values were all greater than 0.93, and the slopes were all greater than 0.90. The precision difference is caused by WRF-Chem simulation, which are mainly caused by the deviation of meteorological field simulation. Nevertheless, the R^2^ in morning hour are all more than 0.80. This could further indicate that our model achieved a stable and robust simulation ability.

Moreover, the station-based 10-CV results were evaluated. Figure S2 shows the station-based 10-CV results from 0800 to 1800 LT in 2018 across the BTH region. Overall, our two-stage model achieved a stable and robust spatial prediction ability. The ensemble station-based 10-CV R^2^ and slope values were 0.90 and 0.89, respectively. Additionally, the RMSE and MAE values were 19.18 and 11.32 μg m^−3^, respectively. This illustrates that our two-stage model can predict the O_3_ concentration accurately in areas with no surface measurement coverage. Furthermore, the station-based 10-CV R^2^ value was slightly lower than that obtained with the sample-based 10-CV approach, which can further indicate the robustness of our model. Similar to the sample-based 10-CV approach, significant diurnal differences in accuracy were also captured with the station-based 10-CV approach. In contrast, more accurate simulation results were obtained from 1200 to 1700 LT, with R^2^ values ranging from 0.89 to 0.90 and slopes ranging from 0.87–0.88. In addition, concentrations with a higher density were distributed close to the 1:1 line. However, a slight underestimation occurred with our model, which could be explained by the simulation uncertainty at the first stage. Despite these limitations, based on our two-stage model, the relationship between the O_3_ concentration and natural and human activities was precisely established.

### 3.2. Station-Scale Accuracy Evaluation

**Figure 3:**
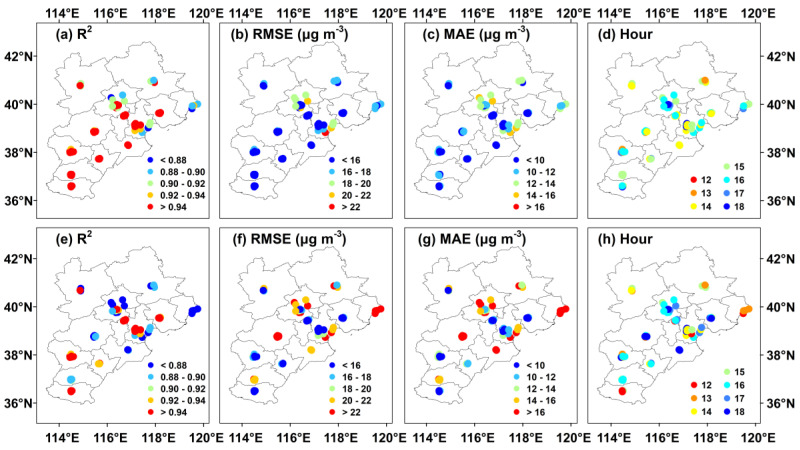
Site-scale evaluation of the estimated hourly O_3_ concentration in 2018 across the BTH region. The upper and lower rows indicate the sample- and station-based 10-CV results, respectively. The columns from left to right indicate R^2^, RMSE, MAE and hour of occurrence of the highest 10-CV R^2^ value.

The performance of our two-stage model in regard to hourly O_3_ concentration estimation at each individual station was also evaluated (Figure 3). In general, our two-stage model attained a high adaptability at each station across the BTH region with a mean sample-based (station-based) 10-CV R^2^ value of 0.95 (0.91) and RMSE and MAE values of 14.25 μg m^−3^ (18.38 μg m^−3^) and 10.20 μg m^−3^ (13.32 μg m^−3^), respectively. Approximately 90% of stations achieved a high accuracy with a sample-based 10-CV R^2^ value higher than 0.90. In contrast, the stations with better O_3_ estimation results were located in the southwestern areas of the BTH region, and the highest sample-based 10-CV R^2^ value could reach 0.98. Furthermore, this model yielded a low uncertainty, and approximately 74% and 68% of all stations attained RMSE and MAE values less than 16 and 10 μg m^−3^, respectively, with the sample-based 10-CV approach. However, the uncertainty in the station-based 10-CV results was slightly higher than that in the sample-based 10-CV results, and at approximately 42% and 31% of all stations, RMSE < 16 μg m^−3^ and MAE < 10 μg m^−3^ were reached in regard to hourly O_3_ concentration estimation. This was mainly attributed to the scattered site distribution and discontinuity in the spatial information, resulting in incorrect assessment of the relationship between the hourly O_3_ concentration and other ancillary data. In addition, we calculated the hour of occurrence of the highest 10-CV R^2^ value of the O_3_ estimates to reflect the hourly adaptive model performance at the station scale. In general, the estimated O_3_ concentrations at each station from 1400 to 1600 LT were the most consistent with the ground measurements, and approximately 79% and 54% of all stations attained the highest sample- and station-based 10-CV R^2^ values, respectively, during this period. This can be interpreted as the stable relationships existing among the temperature, radiation and O_3_ concentration from 1400–1600 LT, suitable for model training. In addition, we selected eight stations, which located in different locations (central, northern, western, eastern, northwestern, northeastern, southwestern, and southeastern regions) of Beijing-Tianjin-Hebei region, to compare the mean hourly simulation results with the observation O_3_ concentration (Figure S3). Overall, the simulation results are completely consistent with the monitoring results, and the correlation coefficients are all more than 0.99.

### 3.3. Temporal-Scale Accuracy Evaluation

First, we evaluated the estimation bias in a time series of the hourly O_3_ concentration from 0800 to 1800 LT across the BTH region (Figure S4). Here, the bias was calculated as the difference between the surface measured and estimated O_3_ concentrations. Overall, the estimation bias indicated a notable diurnal variation involving an initial increase and subsequent decrease. The maximum hourly O_3_ bias was captured at 1600 LT (~0.97 μg m^−3^), while the minimum bias occurred at 1700 LT (~0.06 μg m^−3^). Notably, the biases from 1000–1400 LT were all negative, which indicates that there occurred a slight overestimation with our model during this period. In contrast, the estimation bias from 1500–1800 LT suggested slight underestimation. In addition, the standard deviation of the bias was calculated for each hour, as shown in Figure S4. The hourly standard deviation of the bias remained at a lower level, ranging from 12.70 μg m^−3^ (0900 LT) to 17.89 μg m^−3^ (1800 LT), which is consistent with the O_3_ loading results. This further confirmed the stability of our model for hourly O_3_ concentration estimation.

**Figure 4:**
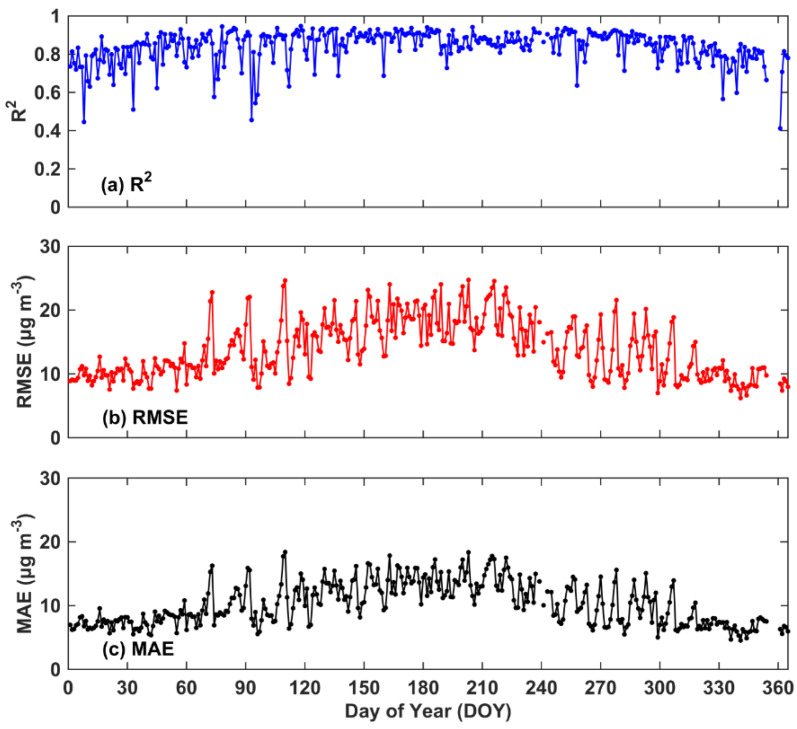
The temporal time series of the consistency between the two-stage model-derived concentrations and surface measurements in 2018 across China. (**a**–**c**) was R^2^, RMSE and MAE, respectively.

We also investigated the estimation performance at the daily scale, and the daily sample-based 10-CV R^2^, RMSE and MAE values for the DOY were all calculated here. Figure 4 shows the temporal performance of our two-stage model. In 2018, the sample-based 10-CV R^2^ value ranged from 0.41 to 0.95, and the average R^2^ value reached approximately 0.84 across the BTH region. Overall, approximately 74.3% of all days in 2018 attained a 10-CV R^2^ value greater than 0.7. However, only three days attained a R^2^ value lower than 0.50 because of the lack of training samples, and the training samples on these days were at least a quarter fewer than those on the other days. Figure 4a also shows that the R^2^ value in the spring and summer was lower than that in the summer. This disparity occurred because the dominant photochemical reaction conditions (e.g., temperature and radiation) in spring and winter are weak, which is adverse to simulating near-surface O_3_ concentration by the WRF-Chem model. RMSE and MAE were low across the BTH region, and approximately 85.8% and 87.1% of all days in 2018 exhibited values less than 20 and 15 μg m^−3^, respectively. High values were mainly captured in summer because of intense photochemical reactions, and severe O_3_ pollution days occurred during this season.

The MDA8 O_3_ concentration is an important standard to evaluate the daily ozone pollution level. Therefore, we also evaluated temporally synthesized MDA8 O_3_ data from hourly samples in 2018 across the BTH region (Figure S5). At the daily scale, compared to surface measurements, our model could accurately reflect daily MDA8 O_3_ variations with a high consistency (R^2^ = 0.96, and slope = 0.94) and low uncertainty (RMSE = 9.84 μg m^−3^, and MAE = 7.23 μg m^−3^). In addition, a significant consistency was captured between the monthly and seasonal mean MDA8 O_3_ concentrations and surface observations, and the R^2^ (slope) value at both scales was 0.98 (0.98). Furthermore, the scatter points were distributed close to the 1:1 line, which could also suggest that our method is reasonable and stable in terms of O_3_ estimation. Moreover, at these two time scales, the estimation uncertainty was reduced, with mean RMSE (MAE) values of 5.50 μg m^−3^ (4.22 μg m^−3^) and 4.69 μg m^−3^ (3.66 μg m^−3^) for the estimated monthly and seasonal MDA8 O_3_ concentrations, respectively. Thus, our two-stage model could accurately describe O_3_ pollution across the BTH region, and the derived full-coverage O_3_ concentration can be widely applied in research on economics, epidemiology, and other related disciplines.

### 3.4. Spatial Distribution of Ozone Pollution in the BTH Region

#### 3.4.1. Diurnal Variations in Ozone

Based on our two-stage model, the full-coverage hourly O_3_ concentration was estimated. Figure S6 shows the spatial distribution of the hourly O_3_ concentration in the BTH region from 0800 to 1800 LT in 2018. Overall, the average hourly O_3_ concentration reached 90.12 ± 5.17 μg m^−3^. Due to the notable limitations of photochemical reaction conditions, O_3_ pollution exhibited significant diurnal variation. From 0800–1800 LT, a low level was captured at sunrise with an O_3_ concentration of ~44.86 ± 9.65 μg m^−3^. Then, with increasing temperature, solar radiation and human activities, the chemical reaction conditions of O_3_ and precursor emissions were both enhanced, resulting in O_3_ pollution, and the peak concentration reached approximately 112.73 ± 9.65 μg m^−3^ at 1500 LT. Severe O_3_ pollution occurred in the BTH region from 1300 to 1700 LT, with its concentrations higher than 100 μg m^−3^. In general, the O_3_ concentrations in the morning (0800–1200 LT) were lower than those in the afternoon (1300–1800 LT), and the O_3_ concentration in the afternoon (~107.93 ± 8.09 μg m^−3^) was 1.57 times that in the morning (~68.74 ± 6.88 μg m^−3^).

In terms of the spatial distribution, approximately 46.5% of all areas was exposed to high O_3_ levels with annual mean hourly O_3_ concentrations higher than 90 μg m^−3^, and these high-O_3_ level areas were mainly located in the southeastern and northwestern parts of the BTH region. However, significant diurnal variations in the spatial distribution occurred in this region. From 0800–1000 LT, high-O_3_ loading areas were mainly concentrated in the northern BTH region, at high altitudes. During this period, there occurred more radiation than in low-altitude regions. Moreover, a large amount of cultivated land and forestland cover the area, enhancing the emission of natural source-derived precursors of photochemical reactions (e.g., VOCs, methane, and terpenes). Subsequently, the high-O_3_ pollution regions were mainly located in the southeastern and northwestern BTH areas. In these areas, human activities contributed a large number of NO_x_ and VOCs. Especially in the southern BTH area, many heavy industrial enterprises are located, resulting in very high anthropogenic emissions. Then, as the day progressed, due to the weakening in human activities, the O_3_ concentrations in high-pollution areas decreased toward sunset (1700–1800 LT). Overall, O_3_ pollution exhibits the spatial distribution characteristics of high levels in the south and low levels in the north throughout the BTH region, which is consistent with the spatial distribution of PM_2.5_ pollution [3]. These results indicated that anthropogenic emissions are one of the primary causes of O_3_ pollution.

#### 3.4.2. Seasonal Variations in Ozone

**Figure 5:**
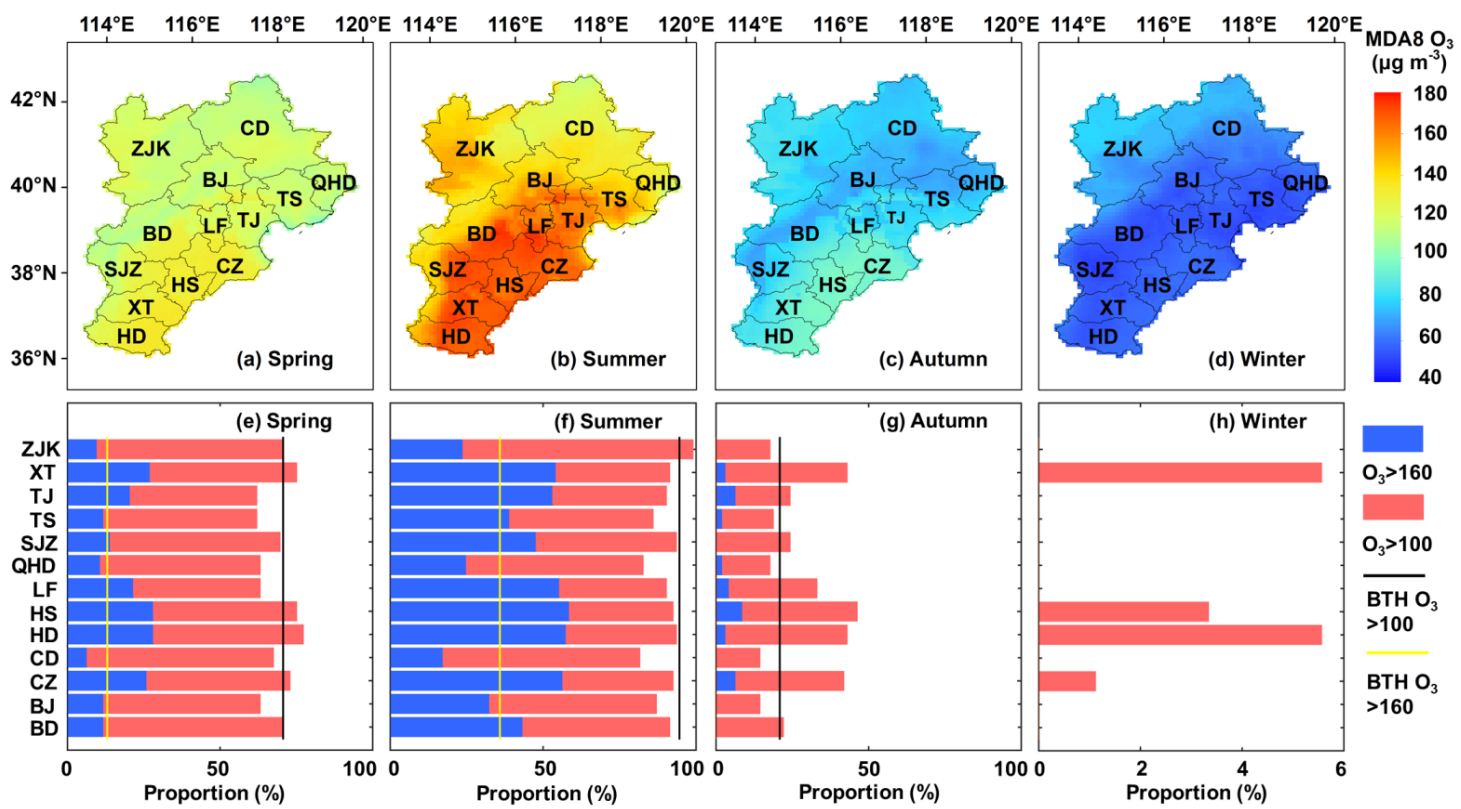
Spatial distributions of the seasonal MDA8 O_3_ concentration (**a**–**d**) and proportion of O_3_ pollution time in 13 cities across the BTH region in 2018: (**a**,**e**) Spring; (**b**,**f**) summer; (**c**,**g**) autumn; and (**d**,**h**) winter.

Due to seasonal subsolar point movement, solar radiation and temperature exhibit notable seasonal variation, resulting in seasonal variations in the O_3_ concentration across the BTH region. Figure 5 shows the spatially average MDA8 O_3_ concentration across the BTH region in 2018, and the seasonal MDA8 O_3_ concentration was synthesized from daily MDA8 O_3_ maps. In general, the O_3_ pollution levels revealed similar spatial patterns between spring and summer. The mean MOD8 O_3_ concentration reached 120.33 ± 7.21 μg m^−3^ in spring. In addition, we calculated the proportion of O_3_ pollution time over 13 cities throughout the BTH region in each season, as shown in Figure 5. The black and yellow vertical lines indicate the proportion of O_3_ pollution time across the whole region. During approximately 70.7% of the spring season, an MDA8 O_3_ concentration exceeding the first-level pollution standard (100 μg m^−3^) was observed throughout the whole region. Among the various areas, Xingtai, Hengshui, Handan and Cangzhou exhibited a longer exposure to ozone pollution in spring. In summer, O_3_ pollution was severe, and the mean MDA8 O_3_ concentration reached approximately 148.28 ± 32.04 μg m^−3^, which far exceeded the first-level pollution standard of the ambient air quality standards in China. In this season, all areas of the BTH region exhibited high O_3_ pollution levels with mean MDA8 O_3_ > 100 μg m^−3^, and 32.0% of all areas attained a mean MDA8 O_3_ concentration notably exceeding the second-level pollution standard (160 μg m^−3^) of the ambient air quality standards. The high-value areas were mainly distributed in the southern and northwestern BTH areas and included most cities in Hebei Province. Across the whole BTH region, during approximately 94.6% of the time, MDA8 O_3_ > 100 μg m^−3^, while during approximately 35.9% of the time, MDA8 O_3_ > 160 μg m^−3^. Among all cities in this region, the Zhangjiakou region attained the highest proportion of pollution time, with MDA8 O_3_ > 100 μg m^−3^ during 98.9% of the time. However, the time of exposure to extremely serious O_3_ pollution (>160 μg m^−3^) time was relatively short in the Zhangjiakou region. Overall, the time of exposure to extremely serious ozone pollution in 9 cities (i.e., Xingtai, Tianjin, Tangshan, Shijiazhuang, Langfang, Hengshui, Handan, Cangzhou and Baoding) was longer than the average level in the BTH region, especially in Hengshui, where MDA8 O_3_ > 160 μg m^−3^ accounted for 58.7% of the summer season. In contrast, the temperature in this area was higher than that in the other areas of the BTH region (Figure S1), suggesting stronger photochemical reaction conditions. Moreover, the anthropogenic emissions of VOCs and NO_x_ in these cities were higher than those in the other cities due to the intense heavy and transportation industries, resulting in the emission of more precursors, which is beneficial for O_3_ generation. In contrast, O_3_ pollution greatly decreased in autumn and winter, and the average MDA8 O_3_ concentration was 81.59 ± 7.81 μg m^−3^ and 61.84 ± 8.09 μg m^−3^, respectively. Especially in winter, the MDA8 O_3_ concentration in the BTH region throughout the winter was lower than 100 μg m^−3^. The main reason is that the temperature and radiation greatly decrease with southward movement of the direct subsolar point. Note that Xingtai, Hengshui, Handan and Cangzhou exhibited O_3_ pollution time proportions ranging from 1.1–5.6% (>100 μg m^−3^). This phenomenon indicated that although photochemical reaction conditions are unfavorable in winter, high precursor emissions (sourced from motor vehicles and heating) could also increase the risk of O_3_ pollution. In addition, significant spatial spillover effects were existed in O_3_ pollution, indicating that the four cities also are threatened by ozone pollution in the surrounding areas.

#### 3.4.3. O_3_ Pollution in the Beijing-Tianjin-Hebei Region

**Figure 6:**
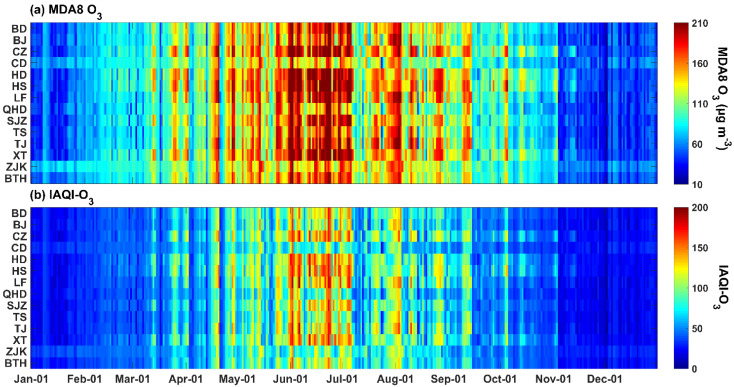
Daily MDA8 O_3_ concentration (**a**) and IAQI of the daily average O_3_ concentration (**b**) in 13 cities and the whole BTH region.

To further explore ozone pollution in the BTH region, we also estimated the daily MDA8 O_3_ concentration in 13 cities (Figure 6). Overall, the daily mean MDA8 O_3_ concentration reached approximately 103.01 ± 43.41 μg m^−3^ across the BTH region. Among the various cities, the highest O_3_ pollution was captured in Hengshui, with a daily MDA8 O_3_ concentration of 113.73 ± 54.98 μg m^−3^. In contrast, the lowest MDA8 O_3_ concentration was captured in Qinhuangdao, with an average concentration of 94.75 ± 43.66 μg m^−3^. In addition, with increasing DOY, the MOA8 O_3_ concentration exhibited a change characteristic of first increasing and then decreasing in all cities, and the MOA8 O_3_ concentration peaked from 1 June to 1 July. Note that O_3_ pollution occurred almost synchronously among the considered cities because of the integrity of the atmospheric transport conditions throughout the BTH region, which illustrates the importance of overall joint governance in this region. Based on the daily MDA8 O_3_ concentration, individual air quality index (IAQI) values of the daily average O_3_ concentration were calculated according to the method proposed by the Technical Regulation on the Ambient Air Quality Index (HJ 633-2012). According to this standard, the IAQI of an MDA8 O_3_ concentration ranging from 0~50, 51~100, 101~150, 151~200, 201~300 and >300 was defined as excellent, good, light pollution, moderate pollution, heavy pollution, and serious pollution, respectively. Similar to the MDA8 O_3_ concentration, variations involving an initial increase and subsequent decrease were also captured for the IAQI. The annual average IAQI value was approximately 58 across the whole BTH region, indicating a good air quality under O_3_ loading. Despite these findings, there remains a long pollution period from May to October. Especially during the period from 1 June to 1 July, moderate O_3_ pollution days were observed.

**Figure 7:**
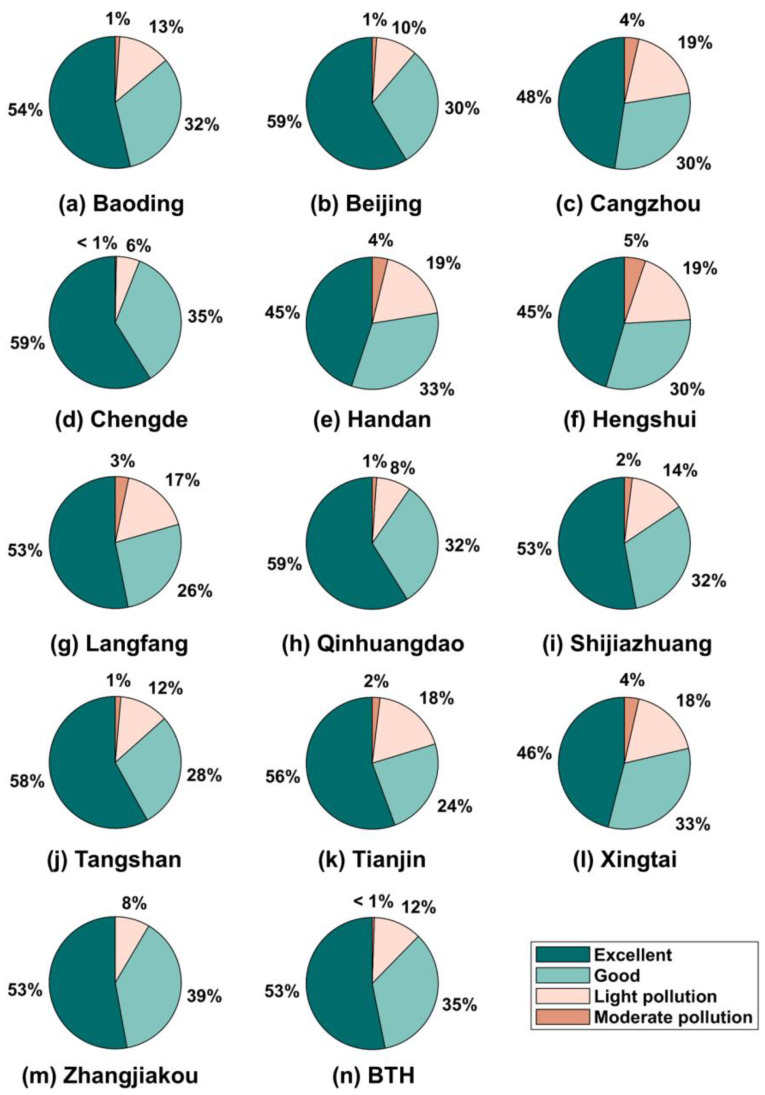
O_3_ pollution level in each city of the BTH region in 2018.

In addition, the proportion of O_3_ pollution days was investigated in this study (Figure 7). Throughout the whole BTH region, there were no severe ozone pollution days, and the excellent and good days of O_3_ level accounted for 53% and 35% in 2018, respectively. However, 12% (43 days) and <1% (2 days) of 2018 exhibited light and moderate O_3_ pollution, respectively. Similar to the seasonal spatial distribution of the MDA8 O_3_ concentration, Beijing, Chengde and Qinhuangdao maintained a low O_3_ pollution level, with days exhibiting an excellent air quality in terms of O_3_ accounting for 59% (215 days) of the year. In contrast, cities with high pollution levels were mainly located in the southern BTH area, with light and moderate pollution days accounting for 17–19% and 1–5%, respectively, of 2018. These results indicated that O_3_ governance is urgently required in the southern BTH area.

## 4. Discussion

Here, we first compared the model performance between our two-stage model and six widely applied traditional models in air pollutant concentration estimation (Table 1). In regard to these models, the same hourly training datasets, except for WRFO_3_, across the BTH region in 2018 were selected for modeling. Among these models, due to the simple linear relationship, the MLR model achieved a poor estimation accuracy with low R^2^ values of 0.63 and 0.62 for the sample- and station-based 10-CV results, respectively. In addition, the RMSE (30.37–31.32 μg m^−3^) and MAE (29.85–31.01 μg m^−3^) values were the highest. Then, since potential nonlinear relationships were captured and spatial relationships were considered, the estimation capability of the GAM and GWR models was higher. The sample-based 10-CV R^2^ values of these two models increased to 0.69 and 0.72, respectively, and the RMSE and MAE values also declined. Furthermore, because fixed and random effects were considered, the estimation capacity of the LME and LME+GWR models was enhanced with sample-based 10-CV R^2^ values of 0.81 and 0.87, respectively. As for using the chemical transport model alone, due to the fixity of its chemical scheme and the particularity of atmospheric transmission, the simulation results are relatively poor. The statistical indicators are even lower than most traditional statistical models with the R^2^ of 0.67 and 0.66 for sample-based and station-based 10-CV, respectively. Meanwhile, the RMSE and MAR of WRF-Chem results alone are also high, which is close to twice that of WRF+RF model. In regard to the machine learning method, we investigated the performance of the RF model. In comparison, with WRFO_3_ input, the coefficient of determination R^2^ increased by 0.03 for both the sample- and station-based 10-CV results. Moreover, the uncertainty (RMSE and MAE) decreased by nearly 10%. These results indicated that our two-stage model is superior to the other traditional models and highlighted the importance and stability of WRFO_3_.

We also compared our results with those obtained with methods adopted in similar studies. Several previous publications are summarized in Table S2 for comparison. Overall, our two-stage model yielded a superior estimation ability than that yielded by previous methods in terms of the various temporal scales. At the hourly scale, compared to the estimation of Liu et al. with a chemical transport model, i.e., the CMAQ model, the CV R^2^ value is at least doubled [58]. Meanwhile, the CV R^2^ value of our model is 0.29 higher than using WRF model alone in BTH region in 2018 [59]. In addition, our model yielded a better sample-based 10-CV R^2^ value than that reported in a previous regional study (R^2^ = 0.81) on the BTH region from 2010 to 2017 with only the RF model for hourly O_3_ estimation [35]. Then, we compared our model to other daily mean or MDA8 O_3_ concentration estimation studies based on machine learning methods, e.g., the data fusion model, XGBoost model and RF algorithm, conducted at the national and regional scales with an approximate horizontal resolution of 0.1° × 0.1° [26,34,36,44]. As indicated in Table S2, the CV R^2^ values are all <0.8 (0.59–0.79), and the RMSE values are all >20 μg m^−3^, which indicates that our model achieves an excellent performance. Furthermore, our two-stage model outperforms many statistical models and machine learning models at the monthly and seasonal scales [33,44]. Although Liu et al. employed the XGBoost model to achieve a high accuracy with R^2^ values of 0.90 and 0.93 at the monthly and seasonal scales, respectively [44], the index values are 0.98 at both scales for our model, which suggests a smaller error than that in the aforementioned studies. Compared with our pervious study, our estimation of near surface O_3_ concentration in Beijing-Tianjin-Hebei region has been slightly improved [60]. In future, we can be extended this method to a wider range.

The purpose of this study was to accurately map the full-coverage hourly O_3_ concentration. However, there remain certain limitations, which will be improved in future research. First, the horizontal resolution can be further improved. Currently, with the needs of refined research in epidemiology, economics and environmental sciences, higher-horizontal resolution high-quality O_3_ maps, e.g., 0.01° × 0.01°, can provide basic data guarantees for more accurate research. Second, the study area should be extended. Here, we only selected the BTH region as an example. Through evaluation, our model achieved an excellent spatial prediction ability, and the model can be widely applied in the estimation of the near-surface O_3_ concentration at the national scale in the future. Third, the time series should also be expanded. The O_3_ monitoring network was established in 2013, and historical records remain unavailable. Therefore, based on the method proposed in this paper, the relationship between the measured O_3_ and other factors can be built since 2013, and the high-accuracy full-coverage hourly O_3_ historical records can be derived over the long term. Furthermore, the WRF-Chem model can predict ozone concentration in future, based on the relationship between the measured O_3_ and other factors, we also can forecast the near surface O_3_ concentration accurately in our future work, which can also be beneficial for research in related fields.

## 5. Conclusions

Currently, the joint management of O_3_ and PM_2.5_ comprises the focus of air pollution control in China. However, high-quality near-surface O_3_ concentration data are relatively scarce in China. This paper attempts to determine the full-coverage hourly near-surface O_3_ concentration, and the BTH region was selected as an example. Therefore, a fusion algorithm (WRF-Chem and RF models) was established that combined meteorological and anthropogenic emission data to estimate the hourly O_3_ concentration in 2018 throughout the BTH region. The assessment results indicated that our model achieved a high accuracy with a sample-based 10-CV R^2^ value of 0.94 and RMSE of 14.58 μg m^−3^. Moreover, the O_3_ concentration estimated with the proposed method was extremely consistent with station-based measurement at varying temporal scales. In addition, after incorporating the chemical transport mechanism and with the use of a data-mining algorithm, the performance of our two-stage model was highly superior to that of the traditional derivation algorithm and methods proposed in previous related studies. With this model, hourly and seasonal O_3_ concentration maps were generated across the BTH region in 2018. The obtained results indicated that the BTH region faces a considerable O_3_ exposure risk with an average hourly O_3_ concentration of 90.12 ± 5.17 μg m^−3^, and the peak concentration reached approximately 112.73 ± 9.65 μg m^−3^ at 1500 LT. Moreover, severe O_3_ pollution mainly occurred in summer. In addition, through calculation of the IAQI associated with the O_3_ concentration, we found that the vast majority of cities suffered slight pollution in 2018 throughout the BTH region, and severe O_3_ pollution regions were observed in the southern BTH area. In summary, the established method is beneficial for accurate O_3_ concentration estimation, and O_3_ maps can be widely applied in economics, epidemiology, and environmental science research.

## Figures and Tables

**Table 1:** Comparison of the model performances between our two-stage model and other widely used traditional model used in air pollutant concentration estimation.

Model	SAMPLE-BASED 10-CV				Station-Based 10-CV			
	R^2^	Slope	RMSE	MAE	R^2^	Slope	RMSE	MAE
MLR	0.63	0.63	30.37	29.85	0.62	0.62	31.32	31.01
GAM	0.69	0.66	27.41	20.06	0.65	0.61	29.57	24.88
GWR	0.72	0.68	25.86	18.43	0.69	0.65	27.50	20.01
LME	0.81	0.79	20.21	14.78	0.79	0.77	22.03	17.27
LME + GWR	0.87	0.85	18.67	13.26	0.85	0.83	21.20	15.11
WRF	0.67	0.69	28.62	22.41	0.65	0.66	29.07	21.74
RF	0.91	0.88	15.84	11.72	0.87	0.85	20.02	14.53
WRF + RF	0.94	0.92	14.58	9.96	0.90	0.89	19.18	13.32

## Data Availability

Not applicable.

## Supplementary Materials

The following supporting information can be downloaded at: https://www.mdpi.com/article/10.3390/ijerph19148511/s1, Figure S1: Spatial distribution of the ground O_3_ monitoring stations in the BTH region; Figure S2: Density scatter plots of the station-based 10-CV results from 08:00 local time (0800 LT) to 18:00 local time (1800 LT) across the BTH region in 2018; Figure S3. The variation of mean hourly measured and simulated ozone concentration in eight stations around Beijing-Tianjin-Hebei region. Figure S4: The time series of hourly O_3_ concentration bias during 0800 LT to 1800 LT over BTH region; Figure S5: Density scatter plots of the sample-based 10-CV results of the daily, monthly and seasonal MDA8 O_3_ concentrations in 2018 across the BTH region; Figure S6: Spatial distributions of the hourly O_3_ concentration from 0800–1800 LT and annual mean O_3_ concentration across the BTH region in 2018; Table S1: VIF, FI and R values between the surface measured O_3_ concentration and all factors for model building; Table S2: Comparison of the model performances between our two-stage model and other model used in other similar studies in O_3_ concentration estimation.
