# Supplementary material for: Hourly Seamless Surface O3 Estimates by Integrating the Chemical Transport and Machine Learning Models in the Beijing-Tianjin-Hebei Region
Source: Int J Environ Res Public Health. 2022 Jul 12;19(14):8511. doi: 10.3390/ijerph19148511 (PMC9324222; doi:10.3390/ijerph19148511)
Supplement: Supplementary file 1 [file ijerph-19-08511-s001.zip › ijerph-1795027-supplementary.pdf]

Supplementary Information for

Hourly Seamless Surface O_3 Estimates by Integrating the Chemical Transport and Machine Learning Models in the Beijing-Tianjin-Hebei Region

Wenhao Xue [1], Jing Zhang [2,*], Xiaomin Hu [2], Zhe Yang [1] and Jing Wei [3,*]

[1] School of Economics, Qingdao University, Qingdao 266071, China
[2] College of Global Change and Earth System Science, Beijing Normal University, Beijing 100875, China
[3] Department of Atmospheric and Oceanic Science, Earth System Science Interdisciplinary Center, University of Maryland, College Park, MD 20742, USA
* Correspondence: jingzhang@bnu.edu.cn (J.Z.); weijing_rs@163.com (J.W.)

Contents of this file

Figure S1-S6

Table S1-S2

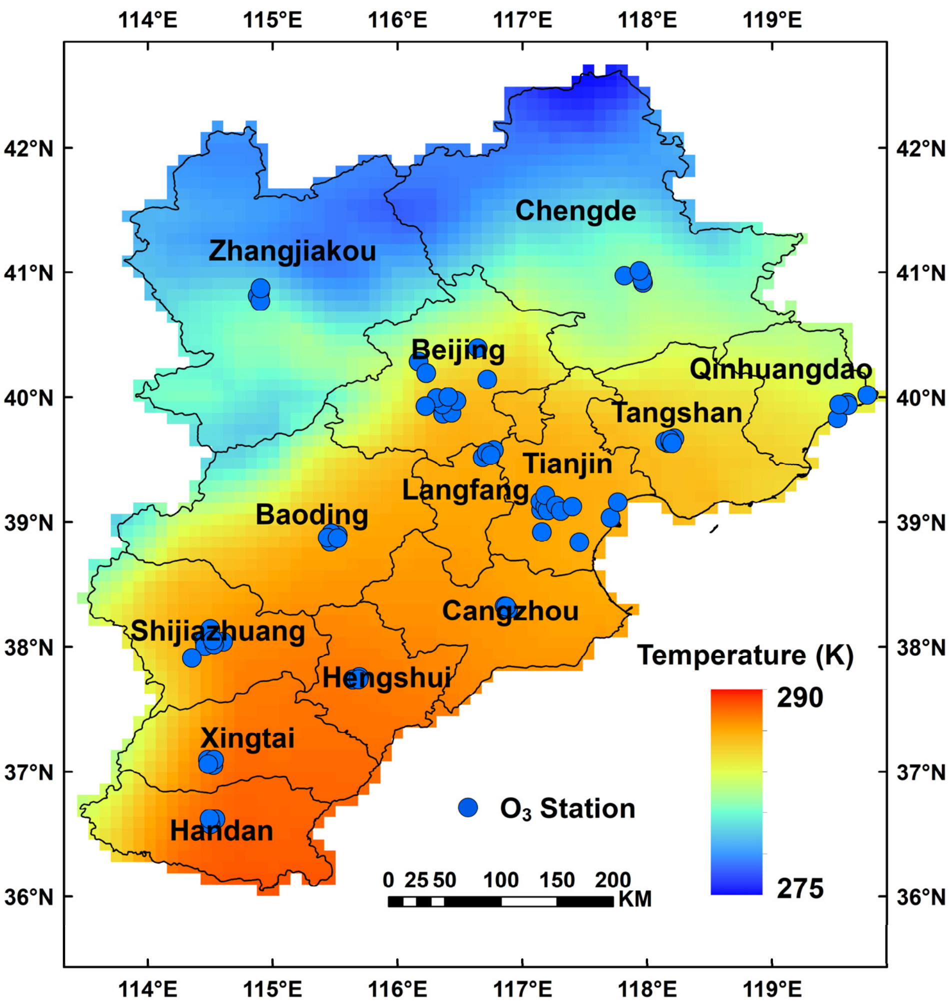

Figure S1. Spatial distribution of the ground O_3 monitoring stations in the BTH region. The background indicates the annual average temperature (unit: K).

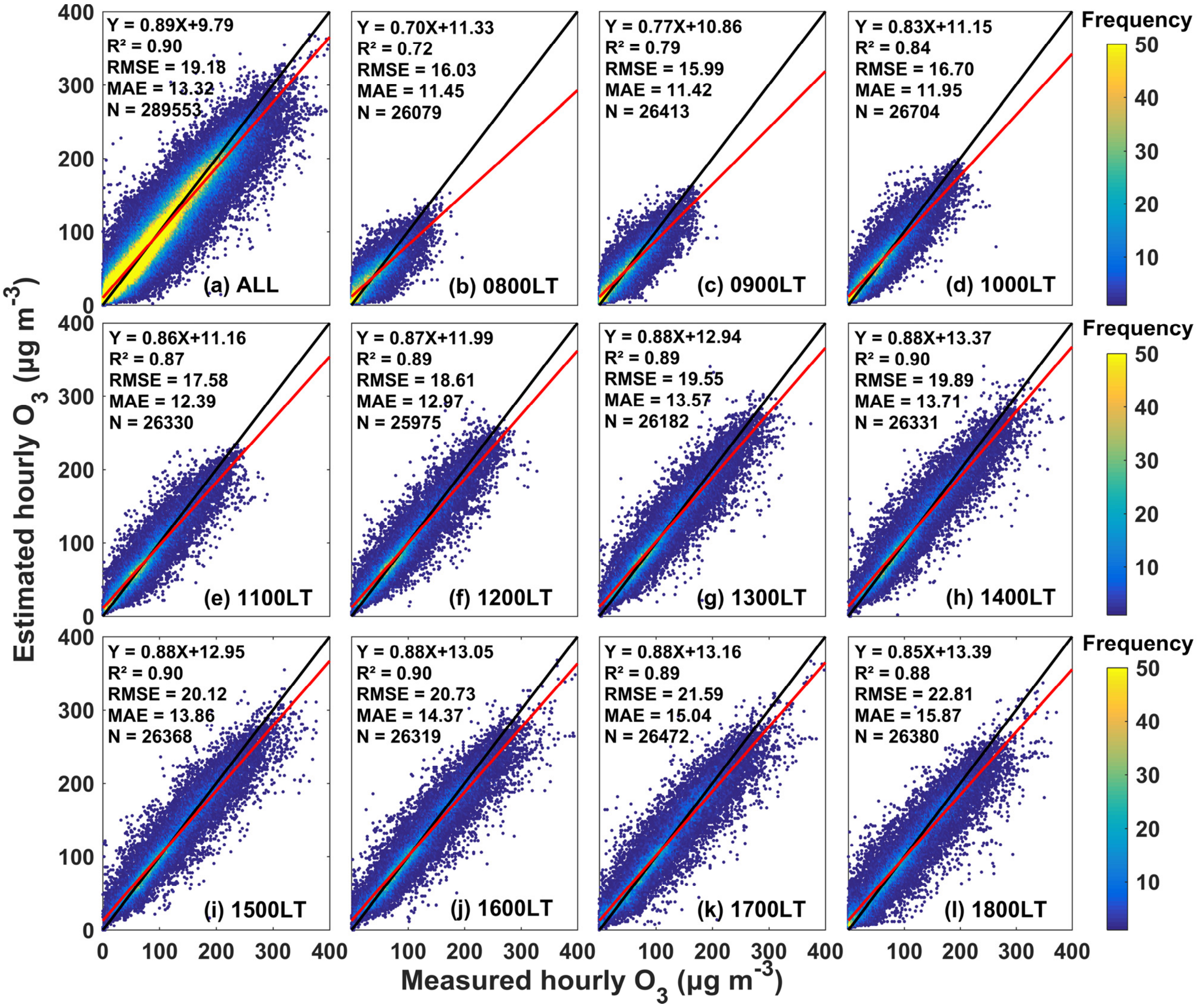

Figure S2. Density scatter plots of the station-based 10-CV results from 08:00 local time (0800 LT) to 18:00 local time (1800 LT) across the BTH region in 2018. (a) All hourly records from 0800 to 1800 LT; (b)–(l) sample-based 10-CV results for each hour from 0800 LT to 1800 LT. The black lines denote 1:1 lines, and the red lines denote linear regression fitting lines.

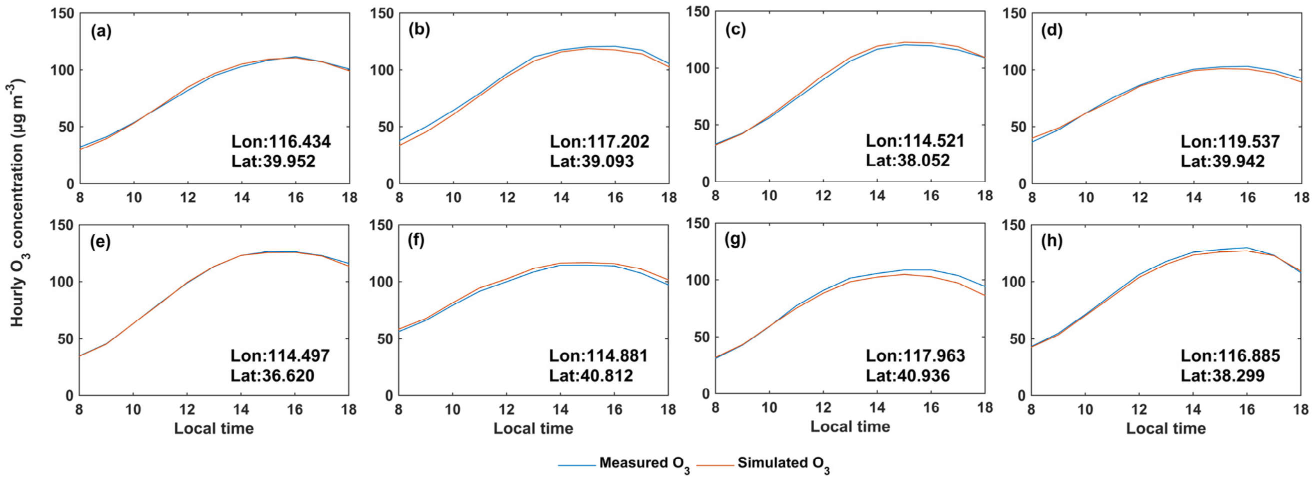

Figure S3. The variation of mean hourly measured and simulated ozone concentration in eight station around Beijing-Tianjin-Hebei region.

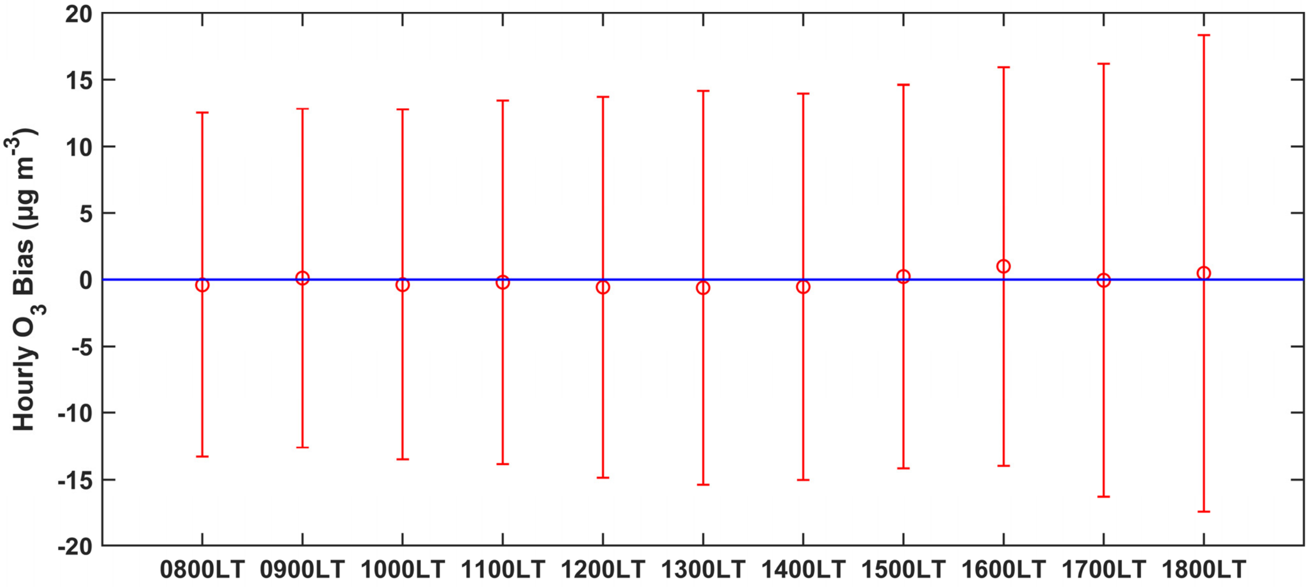

Figure S4. The time series of hourly O_3 concentration bias during 0800LT to 1800LT over BTH region.

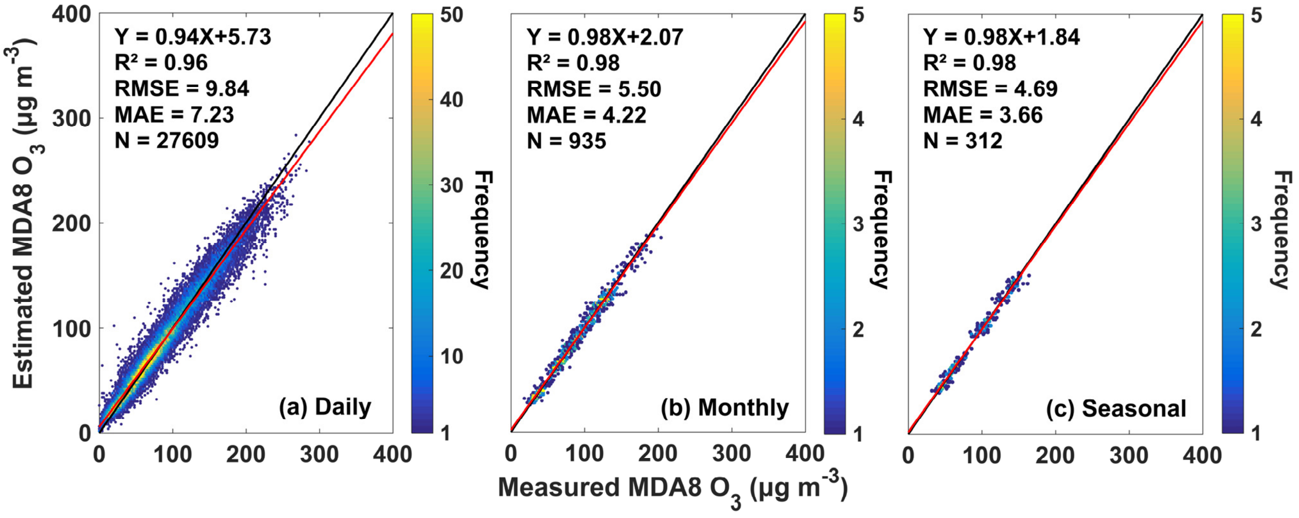

Figure S5. Density scatter plots of the sample-based 10-CV results of the daily (a), monthly (b) and seasonal (c) MDA8 O_3 concentrations in 2018 across the BTH region.

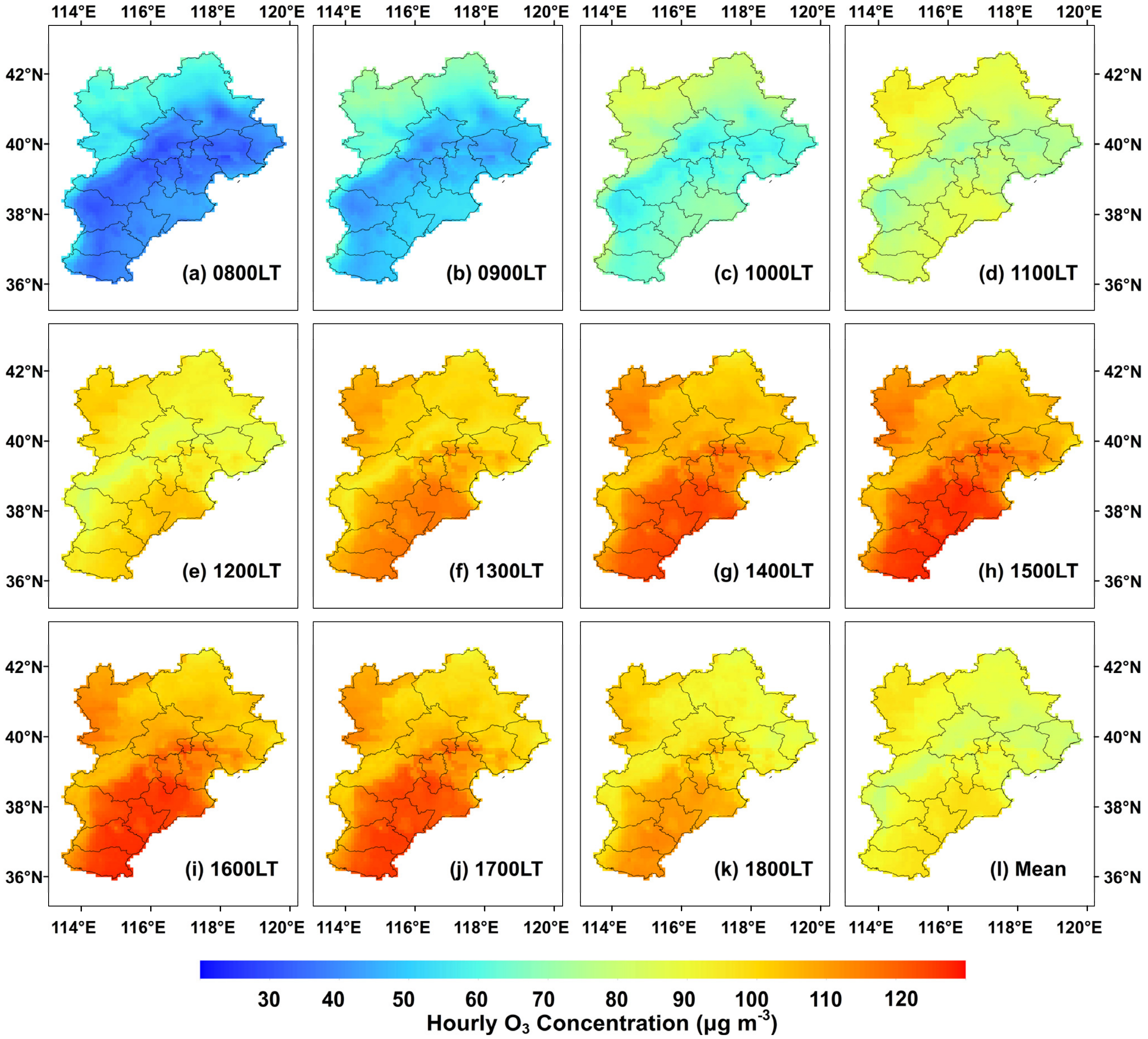

Figure S6. Spatial distributions of the hourly O_3 concentration from 0800–1800 LT (a–k) and annual mean O_3 concentration (l) across the BTH region in 2018.

Table S1. VIF, FI and R values between the surface measured O_3 concentration and all factors for model building.

Variable	$WRFO_3$	TEMI	TEM	RAD	WS	WD	BLH	SP	EVA	RH	CVL	VIDO
R	0.76	-0.08	0.66	0.44	-0.18	0.05	0.42	-0.19	-0.56	-0.08	0.04	-0.40
VIF	1.92	1.35	3.91	2.21	1.27	1.04	2.23	1.19	2.90	1.85	1.13	2.55
FI (%)	59.2	3.1	14.1	6.6	1.8	1.2	2.3	1.7	1.2	5.4	1.6	1.8

Table S2. Comparison of the model performances between our two-stage model and other model used in other similar studies in O_3 concentration estimation.

Method	Region	Spatial resolution	Temporal resolution	Model validation		References
				R^2	RMSE ($\mu g\ m^{-3}$)	
CMAQ	China	36km×36km	Hourly	0.25 – 0.49	19.92 - 41.76	[58]
RF	BTH	0.01°×0.01°	Hourly	0.81	-	[35]
WRF-Chem	BTH	9km×9km	Daily	0.67	38.61	[59]
RF	China	0.1°×0.1°	Daily	0.69	26.00	[34]
Data Fusion Model	China	0.1°×0.1°	Daily	0.70	26.2	[36]
XGBoost	Hainan Island	0.1°×0.1°	Daily	0.59	24.14	[26]
XGBoost	China	0.1°×0.1°	Daily	0.78	21.47	[22]
GWR	Eastern China	0.25°×0.25°	Monthly	0.77	-	[33]
XGBoost	China	0.1°×0.1°	Monthly	0.90	11.09	[22]
XGBoost	China	0.1°×0.1°	Seasonal	0.93	8.32	[22]
STET	BTH	10km×10km	Daily	0.91	18.40	[60]
WRF-Chem + RF	BTH	9km×9km	Hourly	0.94	14.58	Our study
WRF-Chem + RF	BTH	9km×9km	Daily	0.96	9.84	Our study
WRF-Chem + RF	BTH	9km×9km	Monthly	0.98	5.50	Our study
WRF-Chem + RF	BTH	9km×9km	Seasonal	0.98	4.69	Our study
